# A New Symptom in Cerebral Disease

**Published:** 1883-02

**Authors:** 


A NEW SYMPTOM IN CEREBRAL DISEASE.—" In certain morbid conditions," says M. Parrot, " if one pinches the skin smartly, the pupil will be seen to dilate." After detailing two series of cases (in one set of which this symptom was present and in the other absent), he considers the mode of production of the symptom, and he arrives at the opinion that it is not the result of contraction of the radiating fibers through the influence of the sympathetic nerve—this would, in his opinion, be insufficient to overcome the antagonism of the constricting or circular fibers,—but that it results from vascular depletion of the iris owing to constriction of its vessels. In support of this view he reminds us of an observation of Kussmaul's, that the pupil dilates during inspiration and contracts during expiration. According to Parrot the following would be the order of events: irritation of the skin by the pinch, transfer of this irritation to the medullary center by the sensory nerves, its reflection thence to the vaso-constrictors of the iris, depletion of its vessels, dilatation of the pupil. An essential factor in the production of this symptom would be the retention of cutaneous sensibility. In the second series of cases, where this symptom was not present, cutaneous sensibility was nearly, if not quite, lost. He arrives at the following conclusions:—In certain affections of early infancy, with or without convulsions, with or without appreciable lesions of brain, the patient being in a state of persistent coma, if one pinches the skin, a momentary dilatation of the pupil to even two or three times its previous size is produced. Of these affections, those which are characterized by obvious lesion of the nerve-centers are tubercular meningitis, hæmorrhage into the pia mater, certain cases of chronic hydrocephalus, and, lastly, certain ill-defined conditions in which the volume of the brain encroaches upon the cranial capacity. On the other hand, in other morbid states, mostly without convulsions but with coma, the pupil much contracted undergoes no change even when one pinches the skin with sufficient force to produce movement of the face and limbs. In these patients sometimes there exists no appreciable lesion of the nerve centers; at other times there may be œdema or marked congestion ; but in neither case is there any cerebral compression. So far, the only practical conclusion one can draw from this group of facts is the following: a child (affected or not with convulsions) who is comatose, and whose pupils do not dilate when the skin is pinched, is not suffering from meningitis or hæmorrhage into the pia mater, but death is imminent.—(*Revue de Médecine* and *Med. Times and Gaz.* Oct. 1882.)

